# Patterns and indications for quetiapine prescribing in Dutch primary care: a retrospective database study

**DOI:** 10.3399/BJGPO.2024.0219

**Published:** 2025-07-30

**Authors:** Büsra G Cinar, Suzanne A Ligthart, Hugo AJM de Wit, Arnt FA Schellekens, Hanneke HWA Fleuren, Cornelis Kramers, Albert Batalla, Gerard A Kalkman

**Affiliations:** 1 Department of Cinical Pharmacy, Canisius Wilhelmina Hospital, Nijmegen, the Netherlands; 2 Department of Primary and Community Care, Radboud University Medical Center, Nijmegen, the Netherlands; 3 Department of Psychiatry, Radboud University Medical Center, Nijmegen, the Netherlands; 4 Department of Internal Medicine and Pharmacy, Radboud University Medical Center, Nijmegen, the Netherlands; 5 Department of Psychiatry, University Medical Center Utrecht Brain Center, Utrecht, the Netherlands

**Keywords:** Drug prescriptions, quetiapine, antipsychotic drugs, off-label use, retrospective studies, primary health care, Netherlands

## Abstract

**Background:**

Quetiapine, an antipsychotic, is registered for schizophrenia, bipolar disorder, and as an add-on therapy for major depressive disorder. Its anxiolytic and sedative effects make it attractive for off-label uses like insomnia, despite cardiovascular and metabolic side effects. The global increase in quetiapine use over the past decade warrants an examination of its prescribing patterns, especially off-label.

**Aim:**

This study investigated quetiapine prescribing trends in Dutch primary care, with a focus on off-label use.

**Design & setting:**

A retrospective database study using national and regional prescribing data from the Netherlands.

**Method:**

National prescribing trends from 2003–2022 were analysed using data from the Drug Information Project database, focusing on the top 10 antipsychotics. Regional data from the Radboud University Medical Centre (UMC) Technology Centre Health Database provided detailed quetiapine prescribing patterns from 2012–2021, categorised by daily dose. Indications for quetiapine prescriptions from 2020–2022 were derived from the detailed Radboud UMC Technology Centre patient records, including free-text portions, with specific attention for use in sleep problems.

**Results:**

Antipsychotic use increased from 1510 to 2061 per 100 000 population from 2003–2022, largely due to a 13-fold increase in quetiapine (66 to 870 per 100 000 population). Detailed regional data revealed a 3.3-fold increase in quetiapine use from 2012–2021, particularly at doses <100 mg/day. Among new quetiapine users in 2020–2022 from a subset of practices, 76.6% were for off-label indications, and sleep problems were the primary reason for starting quetiapine in 46.9% of cases.

**Conclusion:**

Off-label quetiapine prescribing, particulary for sleep problems, is rising in the Netherlands, despite guideline warnings. Further research on the drivers and long-term effects of this practice is needed.

## How this fits in

Quetiapine prescribing in Dutch primary care has strongly increased over the past decade. Prior data indicated a significant proportion of prescriptions at doses <100 mg/day, suggesting off-label use. Our study shows a continuation of this trend and found that three-quarters of new quetiapine prescriptions were off-label, with sleep problems cited as the primary reason in almost half of all cases. Clinicians should be aware that current Dutch guidelines do not recommend quetiapine for sleep problems due to cardiovascular and metabolic risks and should carefully weigh its risks against any potential benefits.

## Introduction

Quetiapine is a frequently used medication in psychiatry, approved for schizophrenia, bipolar disorder, and as an add-on therapy for major depressive episodes in patients with major depressive disorder who have an inadequate response to antidepressants.^1^ Quetiapine works by binding to histaminergic, adrenergic, serotonergic, and dopaminergic receptors, resulting in anxiolytic and sedative effects in addition to its antipsychotic effect.^1,2^ This pharmacological profile makes quetiapine an attractive option for several off-label indications,^3^ including sleep disorders, delirium, psychosis in Parkinson’s disease, and obsessive compulsive disorder (OCD). For approved indications, the daily maintenance dose of quetiapine is typically 300–800 mg. In contrast, the dosages employed for off-label indications are lower, usually <300 mg.^2^

In the past decade, the global use of antipsychotics, especially quetiapine, has notably increased.^4–8^ Studies have shown a particular increase in low-dose quetiapine prescriptions in several countries, including the Netherlands.^9–11^ For instance, the initiation of low-dose (<100 mg) quetiapine in the Netherlands increased from approximately 10 per 100 000 population to >100 per 100 000 population from 2003–2012.^10^ This increase was observed across both primary care and specialist settings, with most prescriptions given to patients without additional psychiatric medication, suggesting increased off-label use.^10^ In contrast, initiation of regular-dose quetiapine (≥100 mg) increased until 2005, but then stabilised at approximately 50 per 100 000 population.^10^ A similar trend has been observed in Canada, where a 300% increase in quetiapine prescriptions by family physicians was reported from 2005–2012, mostly due to increased prescribing for non-psychotic disorders.^4^

The strong increase in low-dose quetiapine prescriptions might be attributed to off-label use for sleep problems. Research suggests that low-dose (50–150 mg) quetiapine can improve sleep quality in the short term, particularly benefiting individuals with anxiety and depression, but also healthy individuals.^12^ However, quetiapine use is not without risk, even at low doses. Several observational studies have reported associations between low-dose quetiapine use and adverse metabolic or cardiovascular outcomes, including weight gain, metabolic dysregulation, and increased risk of major adverse cardiovascular events.^13–16^ Although these studies generally accounted for potential confounders, there is debate over the extent to which residual confounding might contribute to the observed associations.^17,18^ Pending further high-quality evidence, clinicians should carefully weigh risks against benefits when considering quetiapine for off-label indications. Given these risks, detailed information on current quetiapine prescribing practices, including off-label prescribing, is important. Despite data showing rising trends in quetiapine use until 2012, recent Dutch data to confirm whether this trend has continued are lacking. Additionally, there is limited information on the specific indications for quetiapine prescriptions in the Netherlands.

This study aimed to explore time trends and indications for quetiapine in Dutch primary care. Specifically, it examined 1) time trends in national antipsychotic prescribing practices, including quetiapine; 2) time trends in quetiapine prescribing, in terms of frequency and dosage; and 3) the specific indications for on-label and off-label quetiapine prescriptions.

## Method

### National trends in antipsychotic prescribing

#### Data source

Data on national antipsychotic prescribing trends were obtained from the Dutch Drug Information Project database.^19^ This database is hosted by the Dutch National Health Care Institute, is publicly accessible, covers 95% of all Dutch inhabitants, and contains yearly aggregated data per drug. Drugs can be identified on Anatomical Therapeutic Chemical (ATC) code level.

#### Inclusion/exclusion criteria

Data covering 2003–2022 for all antipsychotics (ATC N05A) were included.

#### Analysis of national trends in antipsychotic prescribing

Data for the top 10 most frequently prescribed antipsychotics in the most recent year (2022) were presented in a figure, including quetiapine, olanzapine, haloperidol, risperidone, aripiprazole, lithium, clozapine, pipamperone, paliperidone, and zuclopenthixol. All remaining antipsychotics were grouped as ’other’. The total number of patients using at least one antipsychotic is shown as ‘total’. Data were normalised to reflect the number of users per 100 000 population using population data from Statistics Netherlands^20^ and plotted against the year of prescription. Since this dataset covered the entire Dutch population, it had no sampling variability. We therefore relied on visual evaluation of the line plots only.

### Detailed regional trends and indications for quetiapine prescribing

#### Data source

Patient-level data on quetiapine prescribing were obtained from the Radboud University Medical Centre (UMC) Technology Centre Health Database. This database contained pseudonymised basic patient characteristics, drug prescriptions, laboratory data, diagnoses, and disease episodes from ~500 000 patients across ~100 general practices in the Netherlands.^21^ Drug prescriptions were coded using the ATC code system, and diagnoses were predominantly coded using the *International Classification of Primary Care, 1st Edition*. A subset of practices, referred to as FaMe-net, used *International Classification of Primary Care, 2nd Edition* and *International Statistical Classification of Diseases and Related Health Problems, 10th Revision* codes. This subset also provided access to the pseudonymised free-text portion of the patients’ medical files, providing detailed information from ~40 000 patients across six practices. The free text was used to assess prescribing indications. In total, 18 adults opted out (rate <0.1%) of sharing their data, which we considered to be too few to distort the results in a meaningful way.

#### Inclusion/exclusion criteria

To investigate trends in dose and frequency in quetiapine prescribing in more detail, all patients in the Radboud UMC Technology Centre Health Database using quetiapine (ATC N05AH04) from 2012–2022 and aged >18 years were included.

Indications for quetiapine prescriptions were determined in patients aged >18 who newly started quetiapine between January 2020 and December 2022. These patients were included from the six practices that provided access to their free-text medical files. New quetiapine use was defined as having no other prescription for quetiapine in the preceding 12 months. Patients with incomplete data (for example, due to being new in the practice) in the 12 months preceding the quetiapine prescription were excluded.

#### Analysis of detailed regional trends and indications for quetiapine prescribing

The number of quetiapine users per year (from 2012–2022) and dose was expressed as the number per 100 000 population, based on the total number of registered patients for all included practices in that year. Patients were classified into three dose categories: <100 mg, 100 to <300 mg, and ≥300 mg total quetiapine dose per day. These categories distinguish between low doses that are more likely to be used for off-label indications and higher doses (≥300 mg), which are in line with on-label indications.^1^ This regional dataset included prescriptions from a large, general population, though were not necessarily representative of the entire country. Because of the large sample size, we relied on visual inspection of line plots only. The indication for quetiapine was derived from the complete medical files, including free text. Indications were classified into one of the following categories: psychotic disorder, bipolar disorder, depression, post-traumatic stress disorder (PTSD), anxiety disorder, panic disorder, personality disorder, OCD, neurodevelopment disorder, psychological complaints, neurasthenia (including burnout), sleep disorder, and other/unknown. This categorisation distinguished between psychological complaints (symptoms without a formal diagnosis) and formal disorders (according to the *Diagnostic and Statistical Manual of Mental Disorders, 5th Edition* classification). Off-label indications were only coded when there were no on-label indications (psychotic disorder, bipolar disorder, and depression) mentioned in the free text. A disorder (for example, panic disorder or anxiety disorder) was only coded if it was specifically mentioned as such. The category psychological complaints was used when no specific disorder was mentioned, for example, *‘anxiety complaints’*, *‘depressive feelings’*, or *‘worrying’*. A primary sleep disorder was chosen if there was no mention of any psychiatric disorder or complaint and quetiapine was explicitly linked to a sleep problem. If the free text provided insufficient information to determine the indication, then disease episode titles, registered *International Classification of Primary Care, 2nd Edition*/*International Statistical Classification of Diseases and Related Health Problems, 10th Revision* codes, and the reason for encounter were also used.

In addition to the primary indication, we documented instances where quetiapine was prescribed for a sleeping problem in the context of another psychiatric disorder or complaint. This was only done when a sleep problem as a symptom was linked to the quetiapine prescription, and no other symptoms were mentioned in relation to quetiapine. For example, free text reporting *‘Has trouble sleeping due to anxiety, prescribing quetiapine’* would be coded as the indication *‘psychological complaints’* and primarily for sleep. Two researchers (BC and GK) independently coded all cases. Disagreements were resolved by consensus. A third researcher (SL, a participating GP from the FaMe-net network) was consulted when consensus could not be reached. Age was defined as the age in years at the initial quetiapine prescription.

The demographic characteristics and the indications for quetiapine from 2020–2022 were analysed using descriptive statistics and are presented in Table 1.

**Table 1.** Characteristics of the study population (2020–2022) and indication for quetiapine prescribing

**Characteristic**	***n* (%)**	
**Gender**		
Man	117 (38.6)	
Woman	186 (61.4)	
**Mean age, years (IQR)**	47 (35–57)	
**Indications**	***n* (%)**	**Sleep problem as main reason for quetiapine use,** ***n* (%)^a^**
Psychotic disorder	10 (3.3)	2 (0.7)
Bipolar disorder	8 (2.6)	1 (0.3)
Depressive disorder	53 (17.5)	20 (6.6)
PTSD	27 (8.9)	10 (3.3)
Anxiety disorder	29 (9.6)	16 (5.3)
Panic disorder	7 (2.3)	2 (0.7)
Personality disorder	12 (4.0)	4 (1.3)
OCD	6 (2.0)	2 (0.7)
Neurodevelopment disorder	9 (3.0)	3 (1.0)
Psychological complaints^b^	69 (22.8)	36 (11.9)
Neurasthenia (including burnout)	8 (2.6)	4 (1.3)
Primary sleep disorder	31 (10.2)	31 (10.2)
Other	34 (11.2)	11 (3.6)
**Total**	**303** (**100**)	**142** (**46.9**)

^a^In these cases, quetiapine was used to address a sleep problem, even if another psychiatric indication was also present. The percentage is the percentage of the total number of patients. ^b^Symptom diagnosis, without a psychiatric diagnosis. IQR = interquartile range. OCD = obsessive compulsive disorder. PTSD = post-traumatic stress disorder.

All analyses were done using RStudio (version 2022.12.0-535).

## Results

### National trends in antipsychotic prescribing

**Figure 1.**
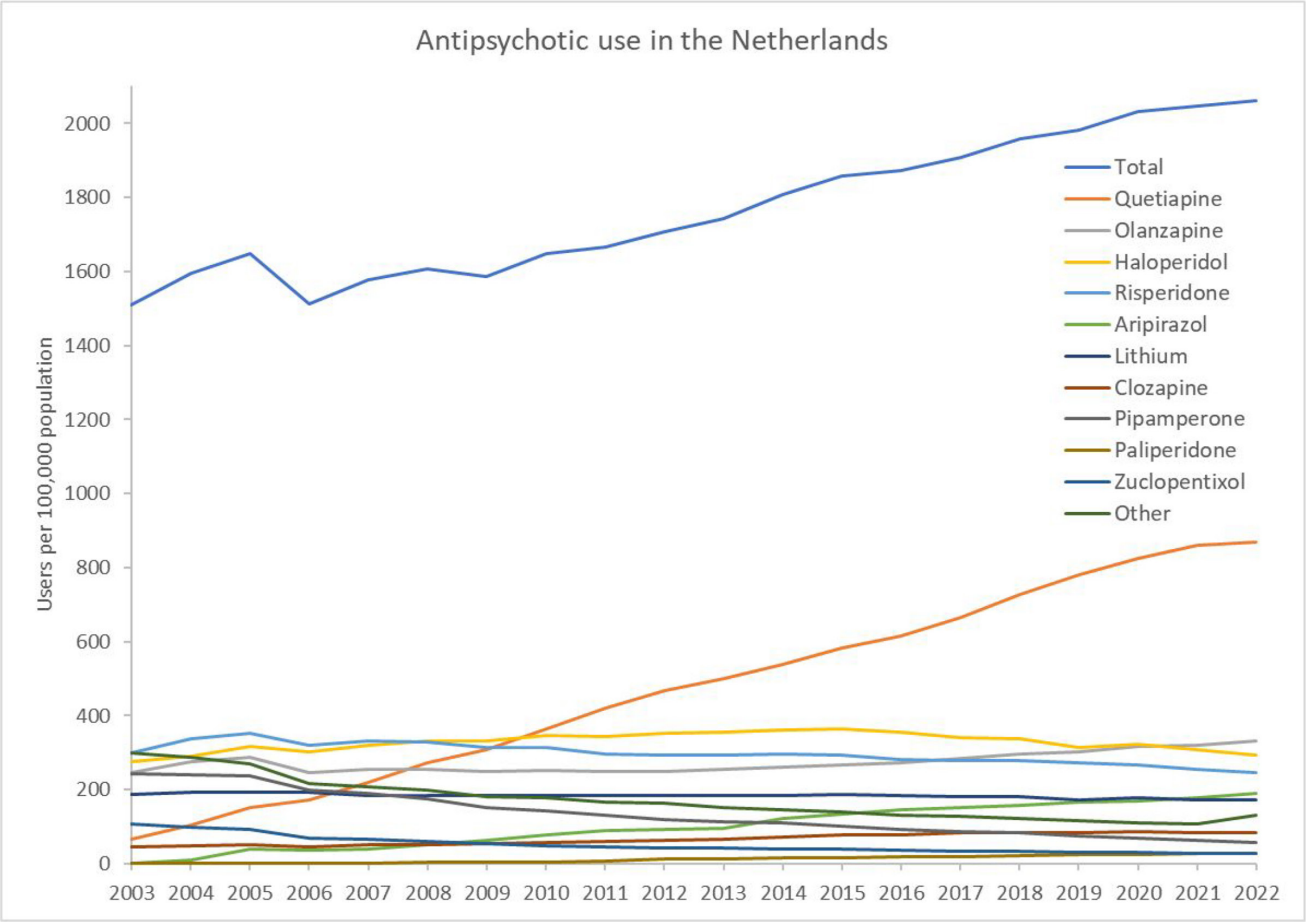
Antipsychotic use per 100 000 population in the Netherlands from 2003–2022

Using the national database, the total number of antipsychotic users remained stable from 2003–2009, and showed an increasing trend from 2009 onward on visual inspection (Figure 1). From 2003–2022 the number of antipsychotic users increased from 1510 to 2061 per 100 000 population. Quetiapine use increased from 66 per 100 000 population in 2003 to 870 per 100 000 population in 2022. In the year before (2021), quetiapine use was 861 users per 100 000 population. In 2010, quetiapine became the most frequently used antipsychotic, with 363 users per 100 000 population. Use of all other antipsychotics remained <400 users per 100 000 population during 2003–2022.

### Detailed regional trends and indications for quetiapine prescribing

**Figure 2.**
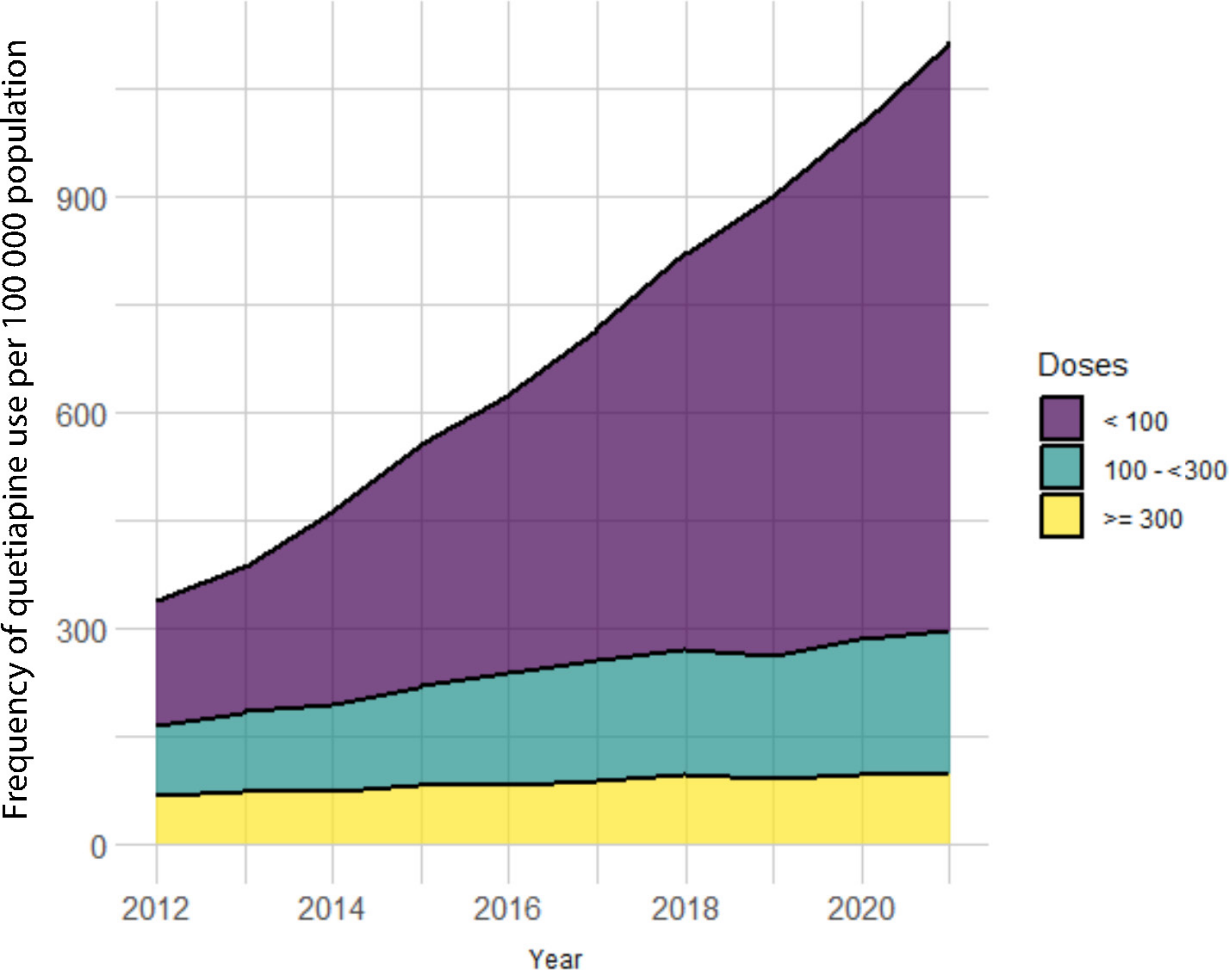
Frequency of quetiapine use per 100 000 population per dosage category (mg) from 2012–2021

In the regional database, a 3.3-fold increase in quetiapine use was seen in the year 2021 compared to 2012. Total quetiapine use increased from 337 users per 100 000 population in 2012 to 1114 per 100 000 population in 2021. Figure 2 shows the number of quetiapine users per dose category per 100 000 population from 2012–2021. Numbers of patients receiving ≥300 mg were stable from 2012–2021. Quetiapine users with a maximum daily dose of 100–<300 mg increased in these years from 97 to 197 per 100 000 population. The largest increase was seen in patients receiving <100 mg quetiapine per day, which increased from 173 per 100 000 population in 2012 to 816 per 100 000 population in 2021.

### Characteristics of the study population 2020–2022

In total, 303 patients met the criteria for determining the reasons for prescribing quetiapine (Table 1). From these patients, 38.6% were men and 61.4% were women. Their average age was 47 years (interquartile range 35–57). Quetiapine was prescribed for the indications shown in Table 1. In this population, 69 patients (22.8%) received quetiapine to treat psychological complaints, such as unhappiness, uneasiness, and stress. In total, 71 patients (23.4%) were prescribed quetiapine for approved indications (in other words, psychotic, bipolar, or depressive disorders). In 46.9% of all patients, a sleeping problem was the target symptom for which quetiapine was prescribed. In 10.2% of the patients, quetiapine was prescribed for a primary sleep disorder, without psychiatric comorbidity.

## Discussion

### Summary

This study investigated patterns for quetiapine prescribing in the Netherlands, using broad nationwide data and more detailed regional data. Nationwide data revealed a strong increase in antipsychotic use from 2003–2021, mostly driven by quetiapine, which increased over 13-fold. Regional data also showed a strong increasing trend, largely driven by prescription of quetiapine at doses <100 mg. Detailed analysis of new quetiapine prescriptions from 2020–2022 confirmed that at least three-quarters of patients were prescribed quetiapine for off-label indications. A sleeping problem was the target symptom or indication for prescribing quetiapine in almost half of all cases.

There were some differences between the national and regional datasets. Although both showed a marked increase, the regional data started lower but later surpassed the national rate (1114 versus 861 per 100 000 population in 2021). Potential explanations include regional differences in clinical practice, demographics (for example, a younger population), and the fact that the regional dataset included prescriptions, whereas national data contained reimbursements.

### Strengths and limitations

The main strength of this study lay in the combination of aggregated national data with detailed regional patient-level information, including free-text portions of medical files, to accurately identify broad trends and provide well-informed interpretations. However, we must also acknowledge a limitation. The reason for prescribing was not always described in the free-text portion of the medical files. Additionally, multiple diagnoses or symptoms were often listed without a clear main reason for prescribing quetiapine. To avoid underreporting on-label use, an on-label indication was chosen when mentioned in the free text. Thus, the high off-label use is unlikely due to mislabeling of indications.

### Comparison with existing literature

Our results aligned with international research showing an increasing trend in off-label quetiapine prescribing.^3,4,8,9,11,22–24^ Several drivers might explain this trend. First, a study among family physicians in Canada found that quetiapine was prescribed off-label to avoid benzodiazepines in patients with complex mental illnesses, out of fear for addiction. Low-dose quetiapine was considered a safe alternative in this population.^25^ Second, a meta-analysis by Grabitz *et al* highlighted that many small, industry-funded studies have suggested quetiapine’s efficacy for off-label uses such as insomnia, PTSD, dementia, and various anxiety disorders.^26^ These exploratory studies potentially foster off-label prescribing and shape clinical guidelines, despite the frequent lack of confirmation in larger trials.^26^ Third, in the Netherlands, changes in benzodiazepine reimbursement policies in 2009, which restricted coverage to specific conditions like epilepsy, anxiety disorders, and palliative care, while excluding insomnia, led to a sharp decline in benzodiazepine use.^27^ Consequently, prescribers may have turned to quetiapine as an alternative. Fourth, a recent survey from Statistics Netherlands reported more sleep problems among adults aged <65 years between 2017 and 2022,^28^ and NEMESIS-3 data indicated a rise in common mental disorders, particularly among younger adults and students, from 17.4% in 2007–2009 to 26.1% in 2019–2022.^29^ These increases in sleep and mental health issues may partially explain the rise in quetiapine prescribing.

Our results showed that quetiapine was often prescribed for sleeping problems, either as a symptom of a psychiatric problem or disorder, or for primary insomnia. A meta-analysis by Lin *et al* ^12^ showed that quetiapine at doses between 50 and 300 mg/day improved sleep quality in patients with psychiatric disorders like general anxiety disorder and major depressive disorder, and primary insomnia. Older patients and males showed the best response. However, the effects were less consistent in healthy subjects and long-term effects remain unclear.^12^ Current Dutch guidelines recommend non-pharmacological treatments, such as cognitive behavioural therapy for insomnia, as the first line of treatment for sleep problems.^30^ Hypnotics, such as benzodiazepines, should only be considered for patients with short-term insomnia that is clearly related to an identifiable cause and associated with a high level of distress. The use of antipsychotics, such as quetiapine, is strongly advised against due to the significant risks of cardiovascular and metabolic side effects.^30^

The off-label use of quetiapine for sleep is particularly concerning given the substantial risks associated with even low doses, including increased weight, blood pressure, cholesterol, and a higher risk of cardiovascular death.^13,16,31^ This practice, possibly driven by the lack of suitable alternatives or the inaccessibility of cognitive behavioural therapy for insomnia, raises critical safety concerns. Although quetiapine can be considered when other treatments are ineffective or unavailable, doctors should be aware of the associated risks and discuss these with patients to ensure they understand the risks of quetiapine before starting therapy. However, the short- and long-term risks of untreated insomnia should also be considered. Untreated insomnia increases the risk of hypertension, depression, cardiovascular disease, and impaired cognitive function, as well as increased accident risk and reduced productivity.^32^ Engaging in shared decision making to balance these risks against the benefits is crucial, especially when quetiapine is not recommended in clinical guidelines for sleep disorders.

### Implications for research and practice

Future research should focus on the effectiveness, tolerance, withdrawal, and negative effects of long-term use of quetiapine for insomnia. Additionally, in light of its widespread use in recent years, it is important to investigate the drivers and rationale behind off-label quetiapine use in primary care. If there is a lack of evidence-based rationale for prescribing off-label quetiapine, policymakers should consider measures to discourage this practice. Moreover, this type of research could expose gaps in current guidelines. Frequent deviations may indicate that certain clinical situations are not adequately covered by existing guidelines. Identifying these gaps can provide new insights that could help improve the current guidelines, ensuring they better address real-world clinical needs.

Our study showed an increasing trend in off-label quetiapine prescription in general practices in the Netherlands, particularly at low doses and primarily for off-label conditions such as sleep problems, despite lacking recommendation in the Dutch clinical guidelines. The frequent use of quetiapine for off-label indications raises serious concerns about patient safety, given the documented risks of cardiovascular and metabolic side effects, even at low doses. It is crucial for prescribers to be aware of the potential risks. While some evidence supports quetiapine’s efficacy for sleep disorders, its use for this purpose is not recommended in clinical guidelines. Furthermore, the evidence supporting many other off-label indications is often limited in scope and duration. Future research should focus on understanding the drivers of off-label use in primary care and evaluating the long-term effects of quetiapine for sleep.

## Data

The dataset relied on in this article is available from the corresponding author on reasonable request.
